# Regulatory Perspectives for AI/ML Implementation in Pharmaceutical GMP Environments

**DOI:** 10.3390/ph18060901

**Published:** 2025-06-16

**Authors:** Sarfaraz K. Niazi

**Affiliations:** College of Pharmacy, University of Illinois, Chicago, IL 60612, USA; sniazi3@uic.edu; Tel.: +1-312-297-0000

**Keywords:** artificial intelligence (AI), machine learning (ML), good manufacturing practice (GMP), pharmaceutical regulation, validation frameworks, digital transformation

## Abstract

Integrating artificial intelligence (AI) and machine learning (ML) into pharmaceutical manufacturing processes holds great promise for enhancing efficiency, product quality, and regulatory compliance. However, implementing good manufacturing practices (GMP) in regulated environments introduces complex challenges related to validation, data integrity, risk management, and regulatory oversight. This review article comprehensively analyzes current regulatory frameworks and guidance for AI/ML in pharmaceutical Good Manufacturing Practice (GMP) settings, identifies gaps and uncertainties, and proposes considerations for future policy development. Emphasis is placed on understanding regulatory expectations across various agencies, including the US FDA, EMA, and MHRA. This article examines verified case studies and pilot programs that demonstrate the successful application of AI/ML under regulatory scrutiny, as well as recent developments in regulatory frameworks and implementation strategies. Ultimately, this article emphasizes the importance of a risk-based life cycle approach and the need for advancements in regulatory science to accommodate the dynamic nature of AI/ML technologies.

## 1. Introduction

Artificial intelligence (AI) and machine learning (ML) technologies are revolutionizing numerous sectors, including healthcare and pharmaceutical manufacturing [1]. In pharmaceutical GMP environments, AI/ML can optimize batch production, enable predictive maintenance, improve process control, and facilitate real-time quality monitoring [2]. Despite these advantages, regulatory agencies remain cautious due to the non-deterministic nature of AI/ML algorithms and the evolving understanding of their risks and limitations [3,4]. The unique characteristics of AI/ML, such as continuous learning and opacity of decision-making processes, pose significant challenges to the principles of GMP, which are grounded in control, reproducibility, and traceability [5].

**Figure 1:**
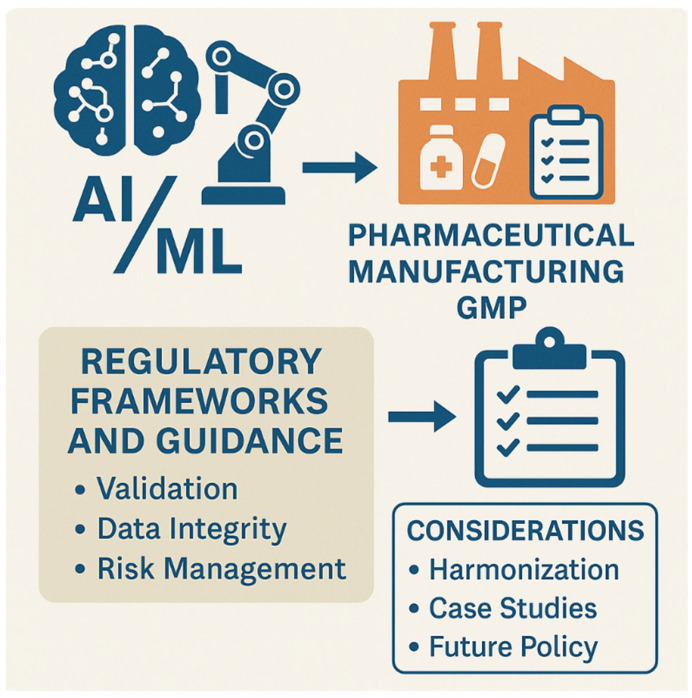
Prospective framework for the AI/ML integration in GMP manufacturing.

The pharmaceutical industry has experienced significant growth in AI/ML adoption, with regulatory submissions containing AI/ML components increasing from a single submission in 2016 to 132 submissions in 2021, representing a substantial five-year growth period [6]. This growth has prompted regulatory agencies to develop comprehensive frameworks that balance innovation, patient safety, and product quality (Figure 1).

## 2. Regulatory Frameworks and Guidance

### 2.1. The United States Food and Drug Administration (FDA)

The FDA has actively engaged with the pharmaceutical industry on AI/ML through discussion papers, public workshops, and guidance documents [7]. The 2019 discussion paper “Proposed Regulatory Framework for Modifications to AI/ML-Based Software as a Medical Device (SaMD)” laid the foundation for a total product lifecycle (TPLC) approach [8]. Although primarily focused on medical devices, its principles apply to AI/ML applications in manufacturing (Table 1).

In 2014, the FDA’s Center for Drug Evaluation and Research (CDER) established the “Emerging Technology Program (ETP)” to assess innovative technologies, including AI/ML in pharmaceutical manufacturing [9]. The program has since evolved into ETP 2.0, providing a structured approach for early engagement between industry and the FDA on novel technologies.

In 2021, the FDA’s Center for Drug Evaluation and Research (CDER) initiated the “Framework for Regulatory Advanced Manufacturing Evaluation (FRAME)” program, which has a specific focus on AI/ML applications in pharmaceutical manufacturing [10]. In March 2023, the FDA published a discussion paper titled “Artificial Intelligence in Drug Manufacturing” as part of the FRAME Initiative, seeking stakeholder input on regulatory considerations for AI implementation [11].

Most significantly, in early 2025, FDA Commissioner Martin A. Makary announced the completion of the first AI-assisted scientific review pilot program and unveiled an aggressive timeline to implement artificial intelligence across all FDA centers by 30 June 2025 [12]. This announcement follows the successful completion of a generative AI pilot program for scientific reviewers, which dramatically improved efficiency, enabling reviewers to complete tasks in minutes that previously took days. Under the leadership of newly appointed Chief AI Officer Jeremy Walsh and Sridhar Mantha, the FDA is prioritizing the immediate deployment of a unified, secure AI system integrated with internal data platforms.

### 2.2. European Medicines Agency (EMA)

The EMA published a reflection paper in 2021 titled “Reflection Paper on Artificial Intelligence (AI) in the Context of the EU Medicines Regulatory Network”, which explores the regulatory implications of AI in the lifecycle of medicinal products [13]. While primarily addressing drug development and pharmacovigilance, it emphasizes that AI applications in manufacturing must comply with Good Manufacturing Practice (GMP) and data integrity standards (Table 2).

Following the implementation of the EU AI Act in 2024, the EMA has been working to clarify implications for pharmaceutical manufacturing [14]. AI systems used in quality control or process control within pharmaceutical manufacturing are generally classified as “high-risk” under the Act, requiring robust risk assessments, human oversight, and transparency measures.

In December 2023, the EMA and the Heads of Medicines Agencies (HMA) published an artificial intelligence work plan for 2028, outlining a collaborative strategy to maximize the benefits of AI while managing associated risks [15]. The work focuses on guidance development, AI tools and technology, regulatory capability building, and international collaboration.

### 2.3. The Medicines and Healthcare Products Regulatory Agency (MHRA)

The MHRA has launched an AI Airlock program to facilitate the safe testing and integration of AI tools within the healthcare sector [16]. In manufacturing, the MHRA supports the development of AI-based quality control systems but mandates comprehensive validation and change control procedures.

The MHRA has demonstrated a progressive approach through its “Innovation Passport” scheme, which includes pathways for advanced manufacturing technologies incorporating AI and machine learning (ML) components. This scheme provides enhanced regulatory support and accelerated assessment for qualifying innovations.

### 2.4. International Council for Harmonisation (ICH)

ICH guidelines provide foundational principles applicable to the deployment of AI/ML in Good Manufacturing Practice (GMP) environments [17]. ICH Q8 (R2), Q9, Q10, and Q11 outline concepts such as quality by design (QbD) and risk management, which are inherently synergistic with AI/ML technologies [18,19]. ICH Q9 (R1) explicitly encourages the use of advanced tools for quality risk management, thereby supporting the use of AI-based predictive modeling within a structured framework [20].

The November 2022 adoption of ICH Q13, “Continuous Manufacturing of Drug Substances and Drug Products”, provides relevant guidance for AI/ML systems used in continuous manufacturing processes [21]. While not explicitly addressing AI/ML, this guideline establishes expectations for process control and monitoring approaches that complement the implementation of AI and machine learning (ML).

### 2.5. The Pharmaceutical Inspection Co-Operation Scheme (PIC/S)

The PIC/S has increasingly addressed AI/ML in its inspector training programs and guidance documents [22]. The organization has been developing guidance on computerized systems in regulated environments, which includes considerations for AI/ML systems. The emphasis is on establishing appropriate governance structures, validating approaches, and conducting ongoing performance monitoring for AI/ML systems used in GMP-critical applications.

## 3. Key Regulatory Challenges

### 3.1. Validation and Verification

One of the central challenges in implementing AI/ML in GMP settings is validating models whose behaviors may change over time [23]. Traditional validation approaches are inadequate for adaptive algorithms. Regulatory authorities advocate for a “locked” model at the time of validation, with a predefined change control plan for any updates. Continuous learning models are viewed skeptically unless robust mechanisms exist for tracking and auditing modifications [24] (Table 3).

Recent developments in validation methodologies have begun to address these limitations [25]. The concept of “dynamic validation” has emerged, involving continuous performance monitoring against pre-established performance metrics, with automated alerts when model drift exceeds acceptable thresholds [26]. The “predetermined change control protocol” (PCCP) methodology provides a structured framework for managing model updates while maintaining regulatory compliance [27,28].

### 3.2. Data Integrity

GMP regulations emphasize ALCOA+ principles—data must be attributable, legible, contemporaneous, original, accurate, complete, consistent, enduring, and available [29,30]. AI/ML systems must be designed to uphold these principles throughout the data pipeline, encompassing the training, testing, and deployment phases. Black-box algorithms can obscure data provenance and undermine auditability, necessitating the implementation of explainable AI (XAI) techniques [31].

With the increasing integration of AI/ML systems with traditional manufacturing execution systems (MES) and laboratory information management systems (LIMS), data lineage has become a critical concern [32]. The “digital thread” concept has gained prominence, referring to the unbroken chain of data relationships from raw material testing through manufacturing to final product release [33,34].

### 3.3. Explainability and Transparency

Explainability is crucial for regulatory acceptance, particularly when AI systems are used in decision-making processes related to product quality and safety [35]. Regulators expect manufacturers to understand the logic behind AI predictions and to provide justification based on scientific and engineering principles. Approaches such as SHAP (SHapley Additive exPlanations) and LIME (Local Interpretable Model-agnostic Explanations) are gaining traction in this context [36,37].

The field of explainable AI (XAI) has advanced significantly, with pharmaceutical-specific applications emerging as a distinct domain [38]. The “Explainability by Design” methodology provides a structured approach to building interpretable models from the ground up, rather than attempting to explain black-box models after they have been developed.

### 3.4. Change Management and Lifecycle Control

AI/ML models evolve, requiring continuous monitoring and a robust change control process. Regulatory agencies expect manufacturers to define a model lifecycle strategy that includes performance monitoring, retraining schedules, version control, and revalidation triggers. Failure to manage these aspects may lead to regulatory non-compliance and product recalls [39].

Recent regulatory discussions have advanced the “progressive validation” concept, which acknowledges the evolutionary nature of AI/ML models and establishes a framework for managing changes within a validated state. This approach defines categories of change with corresponding levels of validation activities [40].

### 3.5. Ethical and Legal Considerations

AI/ML systems may inadvertently introduce biases, especially when trained on non-representative datasets [41]. This can lead to variable product quality or misidentification of out-of-specification events in manufacturing. Regulators emphasize the need for bias detection, fairness evaluation, and mitigation strategies as part of the model validation package.

The legal landscape surrounding AI/ML in regulated environments has undergone significant evolution. The concept of “algorithmic accountability” has been formalized in several jurisdictions, establishing legal responsibilities for organizations deploying AI systems in safety-critical applications [42].

## 4. Implementation Strategies for Compliance

### 4.1. Risk-Based Approach

Regulators are increasingly recommending a risk-based approach to AI/ML implementation, where the level of scrutiny is proportional to the potential impact on product quality and patient safety [43]. Low-risk applications, such as scheduling or maintenance optimization, may require minimal oversight. In contrast, high-risk applications that affect critical process parameters require rigorous validation and documentation (Table 4).

### 4.2. Documentation and Traceability

All aspects of a model’s development, deployment, and maintenance must be thoroughly documented in accordance with Good Manufacturing Practice (GMP) guidelines [44]. This includes the model’s training data, architecture, hyperparameters, performance metrics, and rationale for selection. Version control, change logs, and audit trails are essential to regulatory submissions and inspections.

The industry has made significant progress in standardizing the documentation of AI/ML systems in Good Manufacturing Practice (GMP) environments [45]. Automated documentation tools have emerged to support compliance with these requirements, generating comprehensive documentation packages directly from model development environments.

### 4.3. Human Oversight

AI/ML should augment rather than replace human expertise [46]. Regulatory frameworks stress the role of subject matter experts in overseeing AI predictions, validating outputs, and intervening when anomalies are detected. Hybrid systems that combine human-in-the-loop validation with automated analytics are encouraged as a transitional step.

The concept of “meaningful human oversight” has been refined in recent regulatory communications, distinguishing between different levels of human involvement—from “human-in-the-loop” (decisions that require explicit human approval) to “human-on-the-loop” (where the system operates autonomously but under human supervision) [47].

### 4.4. Model Governance Frameworks

Manufacturers are advised to implement internal AI governance frameworks that include cross-functional representation from quality assurance, data science, IT, and regulatory affairs [48]. Such frameworks should oversee model risk classification, validation strategy, performance monitoring, and compliance audits.

Recent developments in governance practices have included establishing dedicated “AI Quality” functions within pharmaceutical companies, separate from, but working in coordination with, traditional quality assurance departments [49].

## 5. Verified Case Studies and Pilot Programs

### 5.1. Janssen’s Continuous Manufacturing with AI Support

Janssen Pharmaceuticals received FDA approval in 2016 for switching production of its HIV drug Prezista (darunavir) from batch to continuous manufacturing, becoming the first company to receive such approval [50]. While the initial implementation focused on continuous manufacturing processes, subsequent developments have incorporated AI-driven control systems to optimize the process (Table 5).

The success of this initiative demonstrates the feasibility of advanced manufacturing technologies under stringent regulatory requirements. The implementation reduced the testing-to-release time from 30 days to 10 days and enabled better process control through real-time monitoring.

### 5.2. GSK’s Digital Twin Implementation

GSK has pioneered the use of digital twins in pharmaceutical manufacturing through a collaboration with Atos and Siemens, initiated in 2019 [52]. The company successfully developed and implemented digital twins for adjuvant production, representing one of the first applications of a digital twin for a biopharmaceutical process in a GMP environment.

The digital twin system combines computational fluid dynamics (CFD) modeling with machine learning to create a “state estimator model” that maps input process parameters to product quality attributes. The system can operate both offline as a simulator and online to monitor and control manufacturing processes based on real-time sensor data.

Following the successful completion of the proof-of-concept project focused on producing vaccine adjuvant particles, GSK has gradually begun implementing digital twins into its development activities and exploring their applications in R&D upstream processes.

### 5.3. Pfizer’s Vox AI Platform

Pfizer implemented its generative AI platform, Vox, in 2023, marking a comprehensive integration of AI into pharmaceutical operations [51]. Working with AWS cloud services, the platform leverages large language models, including Amazon Bedrock and SageMaker, to optimize manufacturing processes.

According to company reports, AI-powered manufacturing processes have contributed to increased throughput, enabling a faster delivery of medicines to patients. The platform played a crucial role in accelerating COVID-19 vaccine development and manufacturing, with Pfizer’s mRNA prediction algorithm contributing to improved vaccine production efficiency, yielding 20,000 more vaccine doses per batch.

The Pfizer–Amazon Collaboration Team (PACT) initiative has pursued multiple AI projects, including anomaly detection in continuous manufacturing processes using Amazon SageMaker and Lookout for Equipment. Scientists using the platform report potential annual savings of up to 16,000 h in search and data extraction time.

### 5.4. MilliporeSigma’s AIDDISON AI Platform

In December 2023, MilliporeSigma (Burlington, MA, USA) launched AIDDISON™, a software-as-a-service platform that bridges virtual molecule design and real-world manufacturability through AI integration [53]. The platform combines generative AI, machine learning, and computer-aided drug design, trained on over two decades of experimentally validated pharmaceutical research and development datasets.

AIDDISON™ identifies compounds from over 60 billion possibilities that possess key properties of successful drugs, including non-toxicity, solubility, and stability. The platform then proposes optimal synthesis routes, integrating Synthia™ retrosynthesis software through API integration.

The platform has been designed with regulatory compliance considerations from inception, addressing concerns about data integrity, traceability, and validation that are critical for pharmaceutical applications.

### 5.5. Novartis Digital Lighthouse Projects

Novartis has launched multiple AI initiatives in its production facilities through its partnership with Microsoft, announced in 2019 [54]. The collaboration has established an AI innovation lab focusing on leveraging data and AI to transform the discovery, development, and commercialization of medicines.

The “Digital Lighthouse” projects represent Novartis’s strategic digital priority areas, including scaling digital transformation initiatives, enhancing the organization’s digital capabilities, and partnering with technology ecosystems. These projects have included applications in drug discovery, manufacturing optimization, and process control.

## 6. Future Directions and Recommendations

### 6.1. Regulatory Harmonization

Divergent expectations across regulatory agencies create uncertainty for global pharmaceutical manufacturers [55]. Continued efforts are needed to harmonize AI/ML guidance through international forums. The EMA-HMA AI work plan and FDA initiatives represent positive steps toward alignment; however, more coordination is required for consistent global implementation.

### 6.2. Development of AI-Specific Guidance

Current GMP regulations, while applicable, are not specifically tailored to the nuances of AI/ML [56]. The FDA’s planned comprehensive guidance on AI/ML in pharmaceutical manufacturing represents a significant step forward. Similar efforts by other agencies will help provide clarity on model drift, continuous learning, explainability, and algorithmic auditing.

### 6.3. Building Regulatory Capacity

Regulatory agencies continue to invest in developing internal expertise on AI/ML technologies to evaluate submissions and inspect facilities effectively [57]. The FDA’s Technology Modernization Action Plan includes dedicated initiatives for building AI competency among reviewers and inspectors.

### 6.4. Promoting Transparency and Trust

Transparency remains essential for building trust in AI/ML systems [58]. Manufacturers should adopt clear documentation practices and implement robust governance frameworks to ensure transparency and accountability. Industry–regulatory collaboration through existing programs, such as the FDA’s Emerging Technology Program, provides valuable pathways for early engagement and knowledge sharing.

### 6.5. FDA Regulatory Dossier Evaluation Program

On 2 June 2025, the FDA launched Elsa, a generative Artificial Intelligence (AI) tool designed to help employees—from scientific reviewers to investigators—work more efficiently [59]. Built within a high-security GovCloud environment, Elsa provides a secure platform for FDA employees to access internal documents, ensuring that all information remains within the agency. The models do not train on data submitted by regulated industries, safeguarding the sensitive research and data handled by FDA staff. The agency is already utilizing Elsa to expedite clinical protocol reviews, reduce the time required for scientific evaluations, and pinpoint high-priority inspection targets. Elsa is a large language model–powered AI tool designed to assist with reading, writing, and summarizing. It can summarize adverse events to support safety profile assessments, perform faster label comparisons, and generate code to help develop databases for nonclinical applications. These are just a few examples of how Elsa will be used across the enterprise to improve operational efficiency. The introduction of Elsa is the initial step in the FDA’s overall AI journey. As the tool matures, the agency plans to integrate more AI into various processes, including data processing and generative AI functions, to support the FDA’s mission further.

## 7. Conclusions

Integrating AI/ML into pharmaceutical Good Manufacturing Practice (GMP) environments presents both significant opportunities and complex challenges [60]. Current regulatory frameworks provide a foundation for innovation within a risk-based, lifecycle-oriented paradigm; however, continued evolution is necessary as technology and regulatory understanding mature.

Recent developments, including the FDA’s aggressive timeline for implementing AI and the completion of its first AI-assisted review pilot, signal a new era of regulatory acceptance and adoption. However, fundamental challenges, such as validation, data integrity, explainability, and effective governance, remain areas of active development [61].

The verified case studies demonstrate that successful AI/ML implementation in GMP environments is achievable with proper planning, regulatory engagement, and robust quality systems. As the field advances, continued collaboration among industry, regulators, and academia will be essential to developing approaches that enhance product quality and patient safety while enabling innovation [62].

## Figures and Tables

**Table 1:** FDA AI/ML Initiatives and Milestones.

Year	Initiative/Document	Key Focus	Impact
2014	Emerging Technology Program (ETP) Launch	Advanced manufacturing technologies	Assessment framework for novel technologies
2019	AI/ML-Based SaMD Framework	Total product lifecycle approach	Foundation for manufacturing applications
2021	FRAME Program Expansion	AI in manufacturing evaluation	Structured assessment of AI/ML in manufacturing
2023	AI Manufacturing Discussion Paper	Manufacturing-specific AI guidance	Public feedback on AI implementation
2024	CDER AI Council Establishment	Oversight and coordination	Consolidated AI activities across CDER
2025	AI-Assisted Review Pilot Completion	Scientific review efficiency	Agency-wide AI implementation by June 2025

**Table 2:** EMA AI/ML Regulatory Development Timeline.

Year	Initiative/Document	Key Provisions	Regulatory Impact
2021	AI Reflection Paper	GMP compliance requirements	Manufacturing standards alignment
2023	AI Workplan to 2028	Coordinated strategy for AI	Harmonized European approach
2024	EU AI Act Implementation	High-risk AI classification	Robust risk assessment requirements
2024	Final AI Reflection Paper	Updated guidance on AI lifecycle	Enhanced regulatory clarity

**Table 3:** Summary of Key Regulatory Challenges and Solutions.

Challenge Area	Primary Issues	Current Solutions	Regulatory Response
Validation & Verification	Model drift, continuous learning	Dynamic validation methodologies	Enhanced guidance development
Data Integrity	ALCOA+ compliance, data lineage	Digital thread concepts	Enhanced inspection focus
Explainability	Black-box algorithms, transparency	XAI techniques (SHAP, LIME)	Industry best practices
Change Management	Model evolution, version control	Progressive validation approaches	Tiered validation frameworks
Ethical & Legal	Bias detection, algorithmic accountability	Oversight committees	Transparency requirements

**Table 4:** AI/ML Risk Classification Framework for Pharmaceutical Manufacturing. Risk Category Requirements and Examples.

Requirements			
**Complexity Level**	**Low Impact on Product Quality**	**Medium Impact on Product Quality**	**High Impact on Product Quality**
High Complexity	MEDIUM	HIGH	CRITICAL
Medium Complexity	LOW	MEDIUM	HIGH
Low Complexity	MINIMAL	LOW	MEDIUM
**Risk Category**	**Requirements**		**Example Applications**
MINIMAL	Documentation only, basic oversight		Scheduling optimization, inventory management
LOW	Basic validation is required, and routine monitoring		Predictive maintenance alerts, energy optimization
MEDIUM	Enhanced validation + continuous monitoring		Process parameter monitoring, trend analysis
HIGH	Comprehensive validation + regulatory oversight		Real-time quality control, batch release decisions
CRITICAL	Full regulatory pre-approval required		Safety-critical process control, sterile operations

**Table 5:** Summary of Verified AI/ML Implementation Case Studies.

Company/Program	Implementation Year	Key Technologies	Primary Applications	Regulatory Status
Janssen Pharmaceuticals [50]	2016	Continuous manufacturing with AI control	Prezista tablet production	FDA approved
Pfizer Vox Platform [51]	2023	Generative AI, AWS cloud services	Manufacturing optimization, vaccine production	Operational
GSK Digital Twin [52]	2019–2020	CFD modeling, machine learning, Siemens/Atos partnership	Adjuvant production, vaccine development	Proof-of-concept completed
MilliporeSigma AIDDISON [53]	2023	Generative AI, ML, drug design	Drug discovery and synthesis integration	Commercial platform
Novartis Digital Initiatives [54]	2019-ongoing	Microsoft AI partnership, ML platforms	Drug discovery, manufacturing optimization	Multiple programs operational
